# Investigating social drones for recreational running through co-design

**DOI:** 10.3389/frobt.2026.1897014

**Published:** 2026-09-07

**Authors:** Sofia Thunberg, Stine Schmieg Johansen, Martin Bergström, Mohammad Obaid

**Affiliations:** 1 Interaction Design and Software Engineering, Department of Computer Science and Engineering, Chalmers University of Technology and University of Gothenburg, Gothenburg, Sweden; 2 Department of Information Technology, Uppsala University, Uppsala, Sweden; 3 Department of Computer Science, Aalborg University, Aalborg, Denmark

**Keywords:** co-design, human-drone interaction, running, social drones, UAV

## Abstract

**Introduction:**

Human-drone interaction is an emerging field investigating ways to extend the conventionally passive role of drones with autonomous, socially interactive functionalities. Drones offer unique opportunities for applications, such as exercise support, due to their mobility and flexibility. While previous research has studied drones for, e.g., pacing, gait analysis, and accessibility in running contexts, we propose that an equally crucial part of such applications is the socially interactive role of the drone.

**Methods:**

We present a study in two phases. In the first phase, we conducted a preliminary study that informed the design of a co-design workshop through eight semi-structured interviews exploring drone support for runners. In the second phase, we designed and conducted a co-design workshop with five experienced recreational runners to reflect on the practical and ethical challenges of using drones and to envision ways in which drones could support and interact with runners in future scenarios. The collected materials were analyzed using reflexive thematic analysis and annotated visual analysis.

**Results:**

The results present five low-fidelity social drone prototypes, including participants’ reflections tied to each prototype. Furthermore, we present analytical themes that demonstrate the necessity of extending and re-framing approaches to designing social drones.

**Discussion:**

The paper makes three key contributions. First, we offer an approach to co-designing social drones that focus on eliciting the experiences of participants and manifesting those in concrete, embodied design concepts. Second, we validate the existing Design Space for Social Drones framework through a concrete application domain. Our validation shows that the dimensions can be further detailed, and that non-user perspectives are a potentially crucial extension. Third, we present three recommendations that encapsulate concerns for further designs of social drones.

## 1 Introduction

Recreational running is a popular way of exercising. Little preparation and expertise are required, and the potential health benefits are many. It can be difficult, however, to sustain motivation, and runners are at risk of injuring themselves for many reasons, e.g., pushing too hard, running in unfamiliar environments, or changes in the weather conditions. Existing research demonstrates that interactive technologies can have a positive benefit for runners’ motivation (Gute et al., 2022). This includes features such as listening to music, receiving live audio feedback, and competing with other runners. Many interactive technologies to support running exist, such as smartwatches and smartphone applications that can be worn or carried during the run. In addition to technologies that are placed somewhere on the runner’s body, robots have been a focus for supporting and enhancing running. As an example, Puma^1^ demonstrated the use of a mobile ground robot as a pacing device for elite runners during track running. Having an external support device opens a new design space for interactive technologies for running. Certain limitations also arise, particularly for running outside of paved, predictable tracks. While not without limitations, drones (also called ‘Unmanned Aerial Vehicles’ or ‘UAVs’) are versatile robots that can navigate very diverse environments and introduce opportunities both for supporting concrete training goals and for enhancing runners’ wellbeing (Balasubramaniam et al., 2023a).

In parallel to the increasing interest in interactive technologies for running, Human-Drone Interaction (HDI) is growing as a subfield of Human-Robot Interaction (HRI), building on established frameworks of HRI with a focus on how people might engage with autonomous or semi-autonomous flying robotic (drone) platforms. HDI research draws on HRI methods, such as user modeling, proxemic behaviors, and collaboration in teams, and adapts these methods to the unique characteristics of flying robots (drones). These include aspects such as motion dynamics, navigation, and spatial proximity. However, as described in a recent roadmap, ‘the complexity of flight adds new dimensions to be considered, so existing HRI and Human-Computer Interaction (HCI) methodologies need to be adapted for drone research’ Cauchard et al. (2021). Furthermore, as drones become more affordable and accessible, they also become more relevant to everyday contexts, such as sports. Beyond the pervasive use of drones as tools for surveillance, research has also demonstrated the usefulness of drones in social contexts (e.g., Fong et al., 2003). Current commercially available drones have simple physical morphologies in comparison with other robotic agents, and they can mimic behaviors of biological systems, such as bees or ants (Bjurling et al., 2021). Research on social drones focuses on the role of drone morphologies and movement capabilities in social, human-centric contexts (Karjalainen et al., 2017). And like grounded robots, drones can have semi- or fully autonomous behavior, sensing the physical environment and responding accordingly (Baytas et al., 2019). This opens for potential applications across various domains such as entertainment, navigation, companionship, and sports (Obaid et al., 2020a).

In the domain of sports technology, we build upon an existing body of research investigating drone-supported exercise. We specifically select recreational running as our central case study for two key reasons. First, running is a ubiquitous, globally accessible activity requiring minimal resources, making it an ideal, high-impact testing ground for everyday HDI. Second, running presents a uniquely mature subfield within sports-drone literature; prior work has already established the technical viability of drones for pacing (Baldursson et al., 2021), gait analysis (Lafferty et al., 2022), and supporting runners with physical disabilities (Majed et al., 2016). By leveraging a drone’s ability to provide real-time kinetic, kinematic, and spatio-temporal feedback without the constraints of traditional wearables, drone applications can significantly enhance a runner’s health, motivation, and overall enjoyment (Balasubramaniam et al., 2023a).

However, while the benefits of running drones are increasingly investigated, their potential social roles remain largely unexplored. Running is inherently both a deeply personal physical challenge and a highly social activity. Therefore, it serves as the perfect paradigm to investigate how a drone might transition from a mere data-tracking tool into a social companion—either by complementing feedback mechanisms or introducing novel forms of relational support. In response, this work presents a novel approach to designing social drones specifically anchored in running exercise, thereby expanding the broader design space for social HDI.

To aid our investigation, we follow a call for design approaches that involve embodied drones (Baytaş et al., 2019) as opposed to purely conceptual. We present the results from a co-design workshop (Sanders and Stappers, 2008), relying on techniques from the *Social Robot Co-Design Canvases* (Axelsson et al., 2022), where experienced runners produced early design manifestations of drones to support the running practice of collaboratively defined running personas. As a foundation for the workshop, we conducted eight interviews in a preliminary study in which we identified underlying motivations of experienced runners and elicited their reflections about drones. On that basis, we carried out the workshop with five participants with various running experience. The workshop focused on eliciting concrete ways that social drones may support, behave, and interact in specific running scenarios. Our work thus builds on and validates the existing *Design Space for Social Drones* framework (Baytaş et al., 2019) which outlines 12 high-level categories of social drone design, e.g., proxemics, flight, and appeal. The Design Space for Social Drones framework is constructed from a comprehensive literature review, providing a starting point for further research and practice. One crucial direction to strengthen this prior work is to provide further justification of the categories and evaluate the descriptive power of the framework as well as its applicability for specific real-world cases. This is a typical next step to systematically and rigorously assess the utility and soundness of a theoretical framework Fang et al. (2026).

The paper makes three contributions. We present: 1) A co-design approach that encompasses a workshop of four phases (*familiarization*, *brainstorming*, *prototyping*, and *reflections*), resulting in concrete design manifestations of drones, 2) validated and further detailed dimensions of the existing Design Space for Social Drones framework through a concrete application domain, and 3) three recommendations that encapsulate concerns for further designs of social drones.

The paper is structured as follows. We start with a background to sports research in HCI and the specific area of drone support for running exercise in Section 2, followed by discussing previous work on social drone design and ethical considerations for using drones, especially in public spaces. In Section 3, we present a preliminary interview study with eight experienced recreational runner’s that laid the foundation for the co-design workshop. In the following Section 4, we present the method and findings of the co-design workshop. Finally, in Section 5, we present three recommendations for future social drone design and conclude in Section 6.

## 2 Related work

Technologies designed to support running activities can come in many forms and interactions (Peters et al., 2018), such as bands, smartwatches, on-body trackers, smartphone apps, smart insoles, virtual and augmented reality systems, audio-based systems, etc. However, existing running technology comes with certain limitations (Balasubramaniam et al., 2023a). For example, most wearable devices offer retrospective feedback and fail to provide real-time corrective information that could immediately help runners adjust their technique. Similarly, smartphone applications often focus on social engagement or basic metrics rather than detailed biomechanical insights needed for injury prevention and performance improvement. Drone technology is a promising solution to overcome these limitations by providing real-time kinetic, kinematic, and spatio-temporal feedback without requiring runners to wear cumbersome devices, and has the potential of positively impacting runners’ physical health, motivation, and enjoyment (Balasubramaniam et al., 2023a). In recent advancements, drone technologies are being utilized to overcome some of these limitations, since they can sense and track runners from another perspective, and some researchers have proposed concepts utilizing drones in navigating and motivating runners using different interaction modes as illustrated in the next section.

### 2.1 Running with drones

Research on HDI in sports is on the rise, as described by Obaid et al. (2020a) and Baytas et al. (2019). One of the predominant areas of sports that can benefit from the drone’s navigation ability is running, and this has attracted the attention of researchers within the HDI community, including Baldursson et al. (2021), Lafferty et al. (2022), Liao et al. (2021), and Seuter et al. (2018).

For example, Seuter et al. (2018) explored what drone-based services runners desire and how they prefer to control them. An online survey identified desired services like taking photographs, navigation, and safety assistance. An additional study conducted outdoors with runners highlighted a tension between intuitiveness and interference with the running movement, suggesting gesture modulation as a solution (Seuter et al., 2018). Another example, where researchers conducted a study on the use of drones to support recreational runners through pacesetting and video recording for post-run reflection (Balasubramaniam et al., 2023b). Participants found the drone engaging but somewhat unnatural due to drone positioning, speed inconsistencies and noise. Regarding runners preferred real-time feedback provided through drones, one study identified pace, trunk lean, and time as top feedback parameters (Balasubramaniam et al., 2023a).

Studies have also explored how drone technology can enhance the experience of runners and spectators, for example, during amateur races by enabling remote cheering and interaction (Romanowski et al., 2017). Two field studies demonstrated positive user attitudes toward drones, highlighting their potential for improving communication, personalizing experiences, and providing ambient feedback. However, one paper named ‘Filming runners with drones is hard’ sums up the complexity of optimally scheduling drones to film runners during sporting events (Díaz-Báñez and Fabila-Monroy, 2024).

Drone-projected visuals to mediate social running experiences have also been explored, for example, through workshops, developing prototypes, and by testing with potential users (Baldursson et al., 2021). Findings emphasized intuitive visuals reflecting group pace and runner position. Another study used drones to provide visual feedback on runners’ patterns through expressive drone movements, which revealed that drones could effectively increase awareness and motivation, despite some latency and usability issues (Xu, 2024).

Drones have also been used in studies to compare running outdoors and indoors on a treadmill. One study investigated whether traditional indoor treadmill running gait analysis accurately reflects outdoor running conditions using novel drone video technology, showing moderate agreement for foot strike and rear-foot position, but poor agreement for other variables (Lafferty et al., 2022). Finally, one study investigated how drones could help people with physical disabilities by using drones as guides for blind runners (Majed et al., 2016). A pilot test showed participants could accurately locate and follow the drone based on rotor sound alone while being uncertain about running speeds.

The state-of-the-art research suggests that equipping drones with advanced capabilities can offer novel applications, such as using drones to support athletes in running, as illustrated in the above research examples. However, most research have addressing drone technologies for runners have focused on specific interaction modalities between the user and the drone (Obaid et al., 2020a), meaning that the broader needs of runners within the design space of drone technology remain underexplored. This is the core aim of the research presented in this article. Below, we therefore explore the design space of social drones as a potential direction and as a tool for supporting runners.

### 2.2 Social drone design

The concept of a social drone has recently emerged in HDI, referring to drones that entail qualities enabling them to operate socially within human environments across different application domains (Obaid et al., 2020a). This development emphasizes not only the functional capabilities of drones but also their ability to interact with people through social cues and behaviors (Cauchard et al., 2015). Researchers have investigated how users interact with drones in everyday contexts, with particular attention to the verbal and non-verbal behaviors that drones can exhibit or interpret (e.g., Obaid et al., 2020b; Bretin et al., 2025; Yeh et al., 2017; Khamis et al., 2018). These studies, generally, use commercially available drones, which are modified in terms of behavior or physical form to suit specific social tasks or representations.

In contrast, some researchers have focused on the design space of drones specifically for social contexts in inhabited environments (Karjalainen et al., 2017; Johal et al., 2022). Their research explored both the development of individual drone features (e.g., form factors, motion patterns, expressive capabilities) and broader representations of social drone design across different domains. For example, (Karjalainen et al., 2017) conducted a series of three studies to investigate user requirements and the design space of companion drones. They employed participatory design workshops and virtual reality technology to support the design process and to deploy a 3D model of the proposed social drone for testing.

In a literature review on HDI, (Baytas et al., 2019) coined the term *social drones*, referring to drones that operate in human-populated spaces and inevitably engage in some form of social interaction simply by coexisting with people, thus making them inherently social autonomous agents within inhabited environments. In their work, they framed the collated literature around two main perspectives: Drone Design Concerns and Human-Centered Concerns. The drone design concerns include six key aspects: form, flight, control method, sound, lights and display, and proxemics. The human-centered concerns consist of perceived social role, intuitive comprehension, appeal, tactility, intuitive control, and ergonomics. These concerns are context-dependent, some may be more relevant than others depending on the use case, but collectively, they offer a foundational model called *Design Space for Social Drones* for addressing social drone design. In this work, we adopt and build upon these concerns in the context of social drones designed for runners.

Baytas et al. further highlighted a lack of design workshop-based studies and called for future research to develop tools and methods that support the design of autonomous social drones (Baytas et al., 2019). To the best of our knowledge, no research has reported on a co-design approach for developing drone technology in the context of running. This gap has motivated us to take a co-design approach to investigate, understand, and develop design solutions for social drones in the context of running. In this context, we relied on principles from co-design to support participants in imagining and constructing drone concepts for runners. We take inspiration from the framework by (Axelsson et al., 2022) which introduces canvases to guide co-design activities for social robots. We relied on a similar approach to the design process by guiding participants through problem and solution space as well as encouraging collaboration among participants with the aim of prompting diverse perspectives.

### 2.3 Research gap

As described in this section, recent research has established the technical feasibility of drone-supported running through applications such as pacing, gait analysis, navigation, visual feedback, filming, and assistance for runners with disabilities (Majed et al., 2016; Seuter et al., 2018; Baldursson et al., 2021; Lafferty et al., 2022; Balasubramaniam et al., 2023b, Balasubramaniam et al., 2023a; Xu, 2024). In parallel, HDI research has increasingly shifted towards understanding drones as social agents, investigating how drone movement, proxemics, appearance, and social cues influence user perception and interaction (Baytas et al., 2019; Cauchard et al., 2021; Bretin et al., 2025; Lingam et al., 2026b; a). Recent discussions have also identified health, wellbeing, sports, and play as promising application domains for social drones, while emphasizing the need for domain-specific design knowledge and participatory design approaches Baytas et al. (2019); Balasubramaniam et al. (2024).

Despite these advances, little is known about how drones should be designed as social companions for recreational runners rather than as functional sensing or coaching tools. Existing studies primarily evaluate predefined drone interactions, leaving runners’ broader social, motivational, and contextual needs largely unexplored. Moreover, although the Design Space for Social Drones provides a foundational framework for designing socially interactive drones (Baytas et al., 2019), it has not yet been systematically examined within the context of recreational running. This paper addresses these gaps through a participatory co-design study that explores how runners envision socially interactive drones and uses these insights to extend and empirically ground the design space for social drones in sports.

### 2.4 Ethical considerations for drone usage

Prior research has identified a broad set of ethical challenges associated with the design and deployment of autonomous and socially interactive technologies, including drones used in personal fitness and running contexts. Such systems raise concerns that extend beyond technical performance, encompassing regulatory compliance, data governance, and user wellbeing. Regulatory frameworks such as GDPR and the EU AI Act have been discussed as important reference points for ensuring lawful and responsible handling of personal and biometric data. A central theme concerns *privacy and surveillance*. Studies highlight the risk that sensor-rich systems may enable intrusive monitoring or secondary uses of personal data without meaningful consent (Altawy and Youssef, 2016). This concern is particularly relevant in wearable or drone-based monitoring, where continuous data collection can blur the boundaries between assistive functionality and surveillance.

Another line of research examines bias and asymmetrical system performance. Work on biometric and perception technologies demonstrates that uneven accuracy across demographic groups can produce inequitable outcomes (Buolamwini and Gebru, 2018). When applied to motion tracking or running analysis, such disparities could affect the reliability and fairness of feedback provided to users. *Accountability* is also widely discussed in the context of autonomous systems. There is a persistent ambiguity around responsibility when algorithmic systems contribute to harm, unsafe recommendations, or unintended consequences (Novelli et al., 2023). This issue becomes salient for drone-assisted coaching systems that influence user decisions about training intensity or route selection.

HCI research further raises concerns about social deskilling and dependency, where prolonged reliance on intelligent agents may reshape user behavior or reduce engagement with human social support structures (Shevlin, 2024). Related discussions address *exploitative data practices*, where behavioral insights derived from user interaction may be leveraged for persuasive or commercial purposes rather than user benefit. Together, this body of work situates drone-based running assistance within broader ethical debates surrounding autonomous and data-driven technologies, highlighting the need for careful design choices that balance innovation with user rights and social responsibility.

## 3 Preliminary study

A preliminary study was conducted to explore experienced recreational runners’ perspectives on their own running, technology usage for the purpose of running, and how they could envision a drone as running support. Here, we outline the methodology and results from the interviews, followed by the key insights from the preliminary study that informed our design workshop.

### 3.1 Method

Eight semi-structured interviews of approximately 1 hour each were conducted. The interview consisted of 22 questions on the topics of i) participants’ backgrounds, ii) social aspects of their running practices, iii) technologies to support their running and, iv) reflections on drones for running support. Two interviews were conducted remotely via Zoom and six interviews in-person (two in Swedish and six in English). Before each interview, participants provided their informed consent. All interviews were audio recorded.

#### 3.1.1 Participants

The participants (*N* = 8, *mean age* = 49 years old, *SD* = 10.7, 75% men) were recruited through convenience sampling in Gothenburg, Sweden. Six participants were recruited through Parkrun (Parkrun Global, 2025), a global running community that arranges local weekly running meetups. One student and one faculty member at Chalmers University were also recruited. The participants’ years of running experience (*mean* = 18.75 years), and weekly running distance (*mean* = 22.5 km), was also collected (see Table 1 for demographic details).

**TABLE 1:** Interviewed participants. YE = years of running experience; km/w = weekly running distance in kilometers.

ID	Age	Gender	YE	km/w
D1	44	m	25	15
D2	54	f	15	10
D3	63	m	30	15
D4	48	m	30	35
D5	54	m	15	25
D6	47	f	7	35
D7	57	m	15	30
D8	25	m	13	15

#### 3.1.2 Analysis

The audio recordings were transcribed using the AI transcription functionality in Microsoft Word 365 for the English recordings and Klang (Klang AI, 2025) for the Swedish recordings, and the transcriptions were manually checked. An inductive thematic analysis (Braun and Clarke, 2006; Gibbs, 2018) were conducted in Miro, where two of the authors collaboratively coded the transcripts while listening to the interviews. Then, three of the authors identified themes from the codes, and grouped them as follows in the next section. The participants will be referred to as D1-D8, and the quotes are freely translated from Swedish to English when applicable.

### 3.2 Results

Participants emphasized the importance of running for maintaining physical and mental health, as well as avoiding injuries. Many participants enjoyed the challenge of running and were motivated by the desire to improve their performance. Some participants utilized technology during their runs. For them, it was critical that the devices were user-friendly, lightweight, and non-restrictive. Features like a short battery life or cumbersome design were seen as drawbacks. One participant expressed: ‘(The technology) should be made for runners, user-centered and convenient’. (D4). Running was also highly viewed as a social activity, where participants enjoyed group runs or being part of running communities. In the following, we present the two themes: 1) Drones are perceived as tools—not social and 2) imagined expressions from associations.

#### 3.2.1 Drones are perceived as tools—not social

Participants had varied experiences and associations with drones, from observing drones in the sky to piloting drones. Their attitudes towards drones were equally diverse, with some expressing concerns over control and privacy: ‘There was quite a lot of privacy issues over drones flying over people’ (D2). Others highlighted the value of drones in recording situations from unique perspectives. One participant expressed: *‘You can get them to follow, like a skier. It is difficult to capture quality images when skiing, but a drone can make it possible for training purposes’* (D5).

Some participants were reluctant to consider drones as social agents. However, they suggested that with awareness of surroundings, drones could simulate social behavior, for example, by using a GPS-enabled map to navigate and assist runners, identify obstacles, and recommend routes. Additionally, one participant proposed that drones could analyze the user’s running form to prevent injuries or even detect emotional states through camera feeds, enhancing social interaction: ‘So then it could be encouraging you if it senses that you are slowing down or you are getting tired or checking your heart rate or how long you have been running for. And then it gives you encouragement (D6).

#### 3.2.2 Imagined expressions from associations

Initially, participants did not think drones could exhibit behaviors, perceiving them as machines following programmed commands. One participant explained: *‘No, it cannot express anything. You program it to do what you want it to do’* (D5). However, they later acknowledged that certain drone actions, such as movement patterns, proximity to the user, sound, and light, could be used for expressive behavior: ‘I do suppose how it moves … body language. If it is fast and direct, then that is a little bit more aggressive’ (D6).

Participants further reflected that recurring drone behavior patterns could suggest personality traits, such as being extroverted (active and close) or introverted (calmer and distant). One participant compared drone behaviors with how people interpret the behavior of a sports car: *‘Through behavior, how it reacts back. I imagine a drone a bit like a stationary sports car that can make noise—stop and rev. I can imagine that a drone could do that too, making harmonious or aggressive sounds’* (D1).

Participants proposed several methods for interacting with drones, often drawing from their ideas about drone behavior. They suggested communication modalities such as movement, sound, light, and using a smartphone interface. One unique suggestion was adding a cloth tail to the drone, which communicates through wagging patterns: ‘In that case, it must be flying near you. Then it would be a bit more of a dog. Well, why not communicate with a tail?’ (D4).

### 3.3 Key insights

The results provided three key insights, presented with how they informed the co-design workshop:

1) Difficulty to imagine drones for the purpose of running: Because participants struggled to conceptualize drones beyond their current technical limitations, this challenge directly informed our workshop’s scaffolding. To disrupt conventional thinking and spark a creative process, we designed a multi-step progression. Specifically, we introduced custom ideation cards as a generative tool to prompt participants, push them past initial mental blocks, and help them expand their ideas into novel design spaces.

2) Difficulty to imagine social drones: Participants frequently defaulted to viewing drones as purely utilitarian machines or passive data-trackers rather than active agents. To counteract this, we explicitly dedicated structural steps in the workshop to reframe the drone’s agency. We facilitated targeted discussions and designed activities that forced a shift toward the social role of the drone, encouraging participants to conceptualize the technology as an interactive collaborator for recreational running.

3) Ethical challenges with using drones for running: Our interview data surfaced complex ethical concerns, particularly regarding privacy, surveillance, and public space sharing. Rather than treating ethics as an afterthought, these findings led us to bake ethical reflection directly into the workshop. We structured a dedicated, summative phase where participants were required to critically evaluate their own concepts through various ethical lenses, turning abstract concerns into concrete design constraints.

## 4 Co-design workshop

Based on the key insights elicited from the preliminary study, a co-design workshop was conducted to further investigate design choices and implications when imagining social drones for running support. In this section, we first present the method, followed by the findings from the workshop.

### 4.1 Method

The co-design workshop was conducted on campus at Aalborg University in Denmark. It lasted for 2 hours and was conducted in English. Data collection included audio recordings, materials produced through each workshop phase (described below), as well as photographs taken throughout.

**FIGURE 1:**
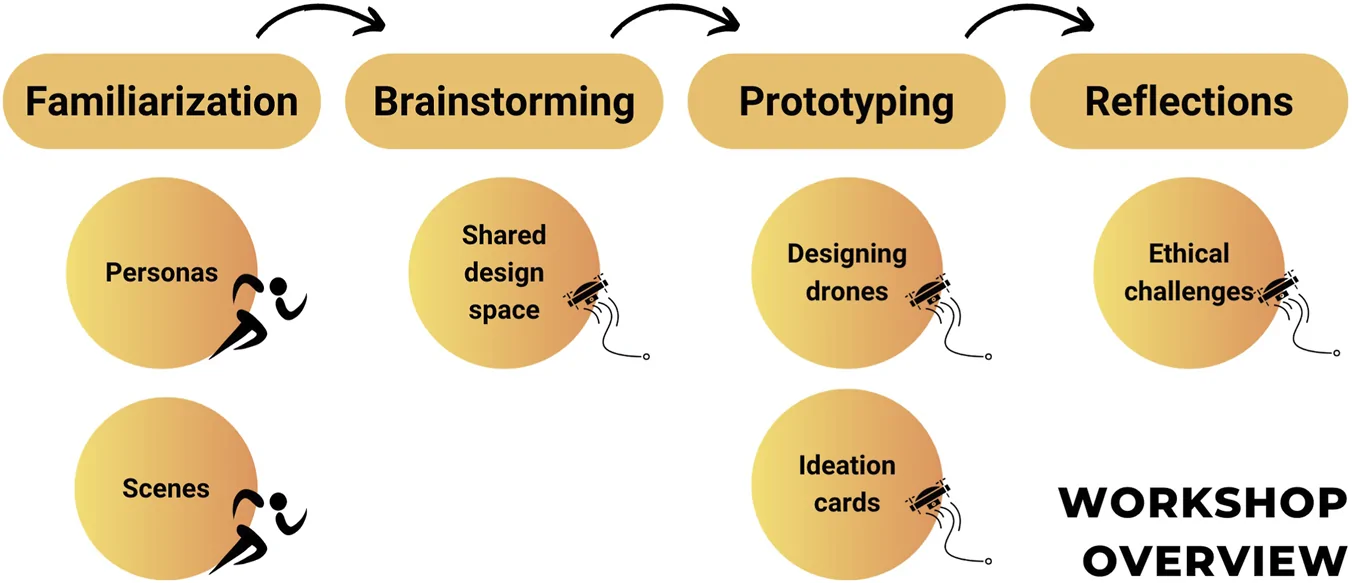
An overview of the phases used in the workshop; familiarization, brainstorming, prototyping, and reflections; covering design considerations of personas, scenes, shared design space, designing drones, ideation cards, and ethical challenges.

The co-design workshop was divided into four phases to elicit creative thinking: 1) *familiarization*, 2) *brainstorming*, 3) *prototyping*, and 4) *reflections* (see Figure 1 for an overview). For each of these phases, two workshop facilitators provided material to structure and constrain the design space as imagined by participants. This is especially crucial when participants are not designers and/or do not have previous knowledge of design processes. The participants were encouraged to think beyond technical feasibility and the shapes of drones they might have already come across. This section provides the overall purpose of each phase, the materials used to facilitate it, and the actual procedure as it unfolded during the workshop.

#### 4.1.1 Participants

Participants (*N* = 5, *mean age* = 31 years old, *SD* = 7.16, 60% men) were recruited through local running clubs on social media and were offered a gift card of 40 USD as compensation. Following our emphasis on rich contextual information (Caine, 2016; Gibbs, 2018), we recruited three participants with few years (2–5) of experience and two participants with many years (15) of experience as well as diverse weekly running distances. Five participants allow for this diversity while still allowing room to discuss particularities of each participant’s experiences (Gibbs, 2018).

We collected informed written consent, participants’ demographic data (age, gender, years of running experience, and weekly running distance), and their previous experiences with running technology and drones, see Table 2. All of the participants regularly used assistive technologies for their runs, such as smart watches, music, and tracking applications (e.g., Strava, Garmin Connect, Apple Fitness).

**TABLE 2:** Participant data. YE = years of running experience; km/w = weekly running distance in kilometers; RT = running with technology (yes/no); OD = have observed drones (yes/no); FD = have controlled a flying drone (yes/no); DL = has a drone license.

ID	Age	Gender	YE	km/w	RT	OD	FD	DL
P1	21	f	2	30	y	y	n	n
P2	26	f	5	20	y	y	n	n
P3	32	m	2	25	y	y	n	n
P4	42	m	15	80	y	y	n	n
P5	34	m	15	10	y	y	y	n

#### 4.1.2 Familiarization

This phase laid a foundation to ground the following phases through the creation of distinct runner personas. Participants were asked to collaboratively develop five runner personas to help them discuss needs, struggles, and ideal runner experiences/aims. We prompted participants to use a persona template (Tomitsch et al., 2020) and imagine runners other than themselves to draw from their own experiences while focusing on new opportunities for the personas. At the end of this phase, each persona was assigned a LEGO figure as a physical representation during the following phases. After developing the personas, participants matched the personas to scenes where they would be running, using seven different photos of running environments (track, forest, city, park, beach, snow, trail). The familiarization phase allowed participants to immerse in environments that the personas might be involved with to aid in later phases where they would speculate about drone usage in those spaces.

#### 4.1.3 Brainstorming

**FIGURE 2:**
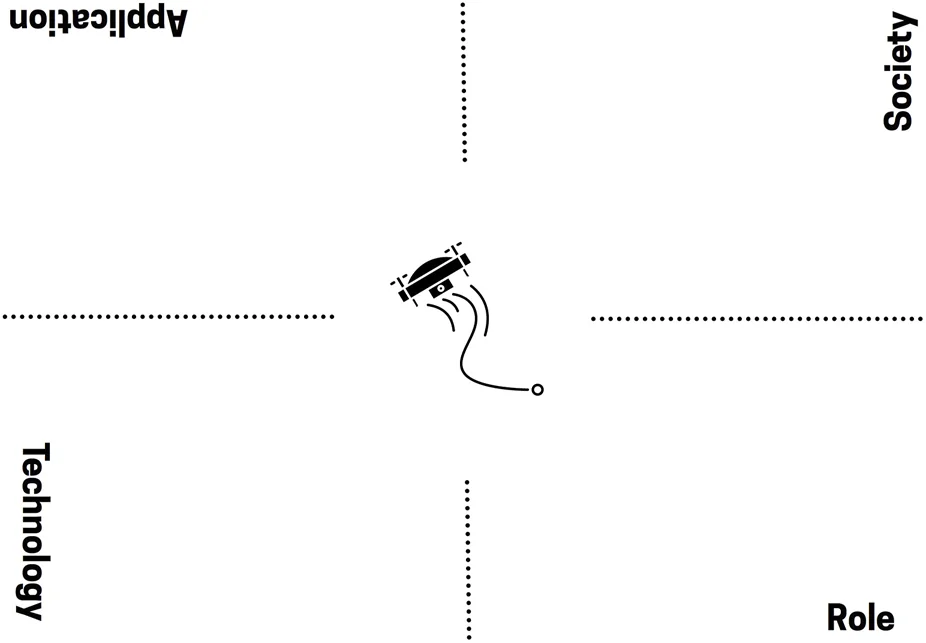
The shared design space, presenting a drone in the middle with four aspects to design by: technology, application, society and role. Previously developed by (Hendriks et al., 2024) and inspired by (Lee et al., 2012).

**FIGURE 3:**
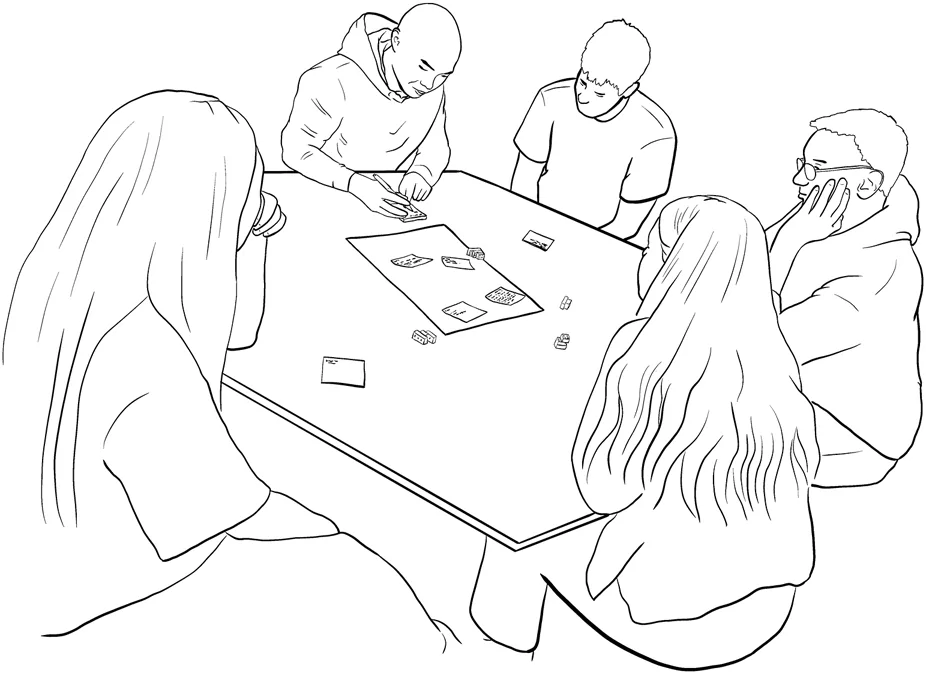
An illustration of the in-person co-design workshop with experienced runners during the brainstorming phase.

This phase aimed to initiate reflections on the concept of a social drone to assist the personas with running. To facilitate brainstorming, we provided a shared design space sheet (see Figure 2), inspired by (Hendriks et al., 2024), where the participants reflected on *technology* (that the drone had), *application* (what the drone helped with), *society* (societal impact of the drone system), and *role* (the social role of the drone). The participants used the LEGO figure while discussing the personas and attached post it notes too the design space (see Figure 3 for an illustration).

#### 4.1.4 Prototyping

**FIGURE 4:**
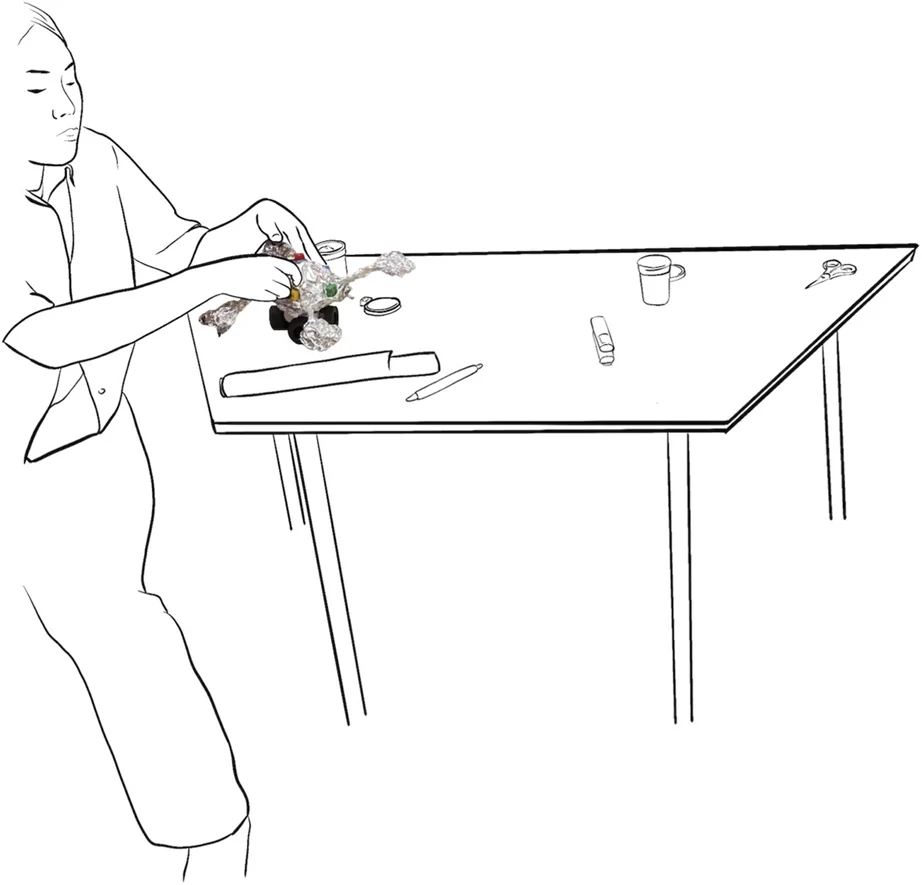
In the prototyping phase, participants turned the conceptual ideas into concrete designs through physical materials.

This phase, see Figure 4, aimed to put the conceptual discussion into concrete design with no focus on technical feasibility. Participants were asked to build a concept of a drone to assist one persona, individually. The participants chose one persona each and designed a drone for that persona, resulting in five different designs. Participants could freely choose provided materials, including LEGO, textiles, play dough, paper, aluminum foil, cardboard, tape, scissors, and pens.

**FIGURE 5:**
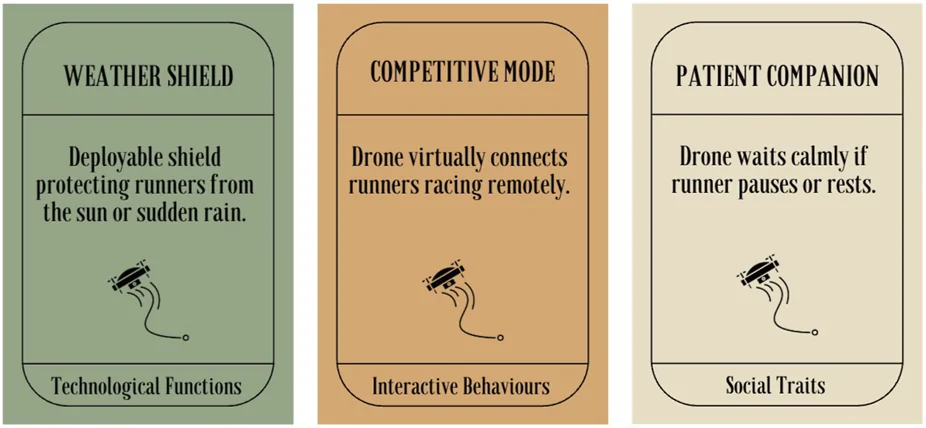
Example of ideation cards. From left to right: technological functions, interactive behaviors, and social traits.

As inspiration while designing the prototypes and to challenge their imaginations of what a drone could assist with, we introduced three sets of ideation cards (see Figure 5). The card structure was inspired by existing design cards for other domains, including the PLEX cards for designing for playfulness (Lucero and Arrasvuori, 2013) and the MeCaMInD toolbox for designing for movements (Elbæk et al., 2023). The 42 cards (see Supplementary Material) presented elements in three distinct categories; 1) *Technological functions* (16 cards), 2) *Interactive behaviors* (15 cards), and 3) *Social traits* (11 cards), and they consisted of a name (e.g., ‘Weather Shield’) and a short description (e.g., ‘Deployable shield protecting runners from the sun or sudden rain’). Each category of ideation cards was introduced to the participants one by one, starting with technological functions, followed by interactive behaviors, and, finally, social traits. The total time for the prototyping phase was 40 min, with one category of cards introduced every 10 min. At the end of the phase, participants presented their designs to each other.

#### 4.1.5 Reflections

This phase aimed for participants to step back from concrete designs to discuss the implications of their creations. Five ethical challenge cards were formulated from the results of the preliminary study and related work on ethical considerations for drone usage (see Section 2.4 and 3). Each challenge was presented sequentially, where each featured a heading, for example, *Privacy and Surveillance*, a quote from the interviews to exemplify the challenge (e.g., ‘Considering science fiction films, engaging in conversation with artificial intelligence does not seem unusual at all. In fact, I do not perceive any real barriers preventing such interactions.’, and example questions, for example, ‘Should drones collect personal data to support running even though there is a risk of data breaches?’. The participants discussed the challenge in relation to the drone designs and ranked the drones from the most to the least challenging design in regard to the specific challenge.

#### 4.1.6 Post-workshop survey

Following the workshop, a short evaluation survey of the method was conducted. In the survey, we asked participants to rank the elements they found most helpful to elicit ideas and reflections about the drones.

#### 4.1.7 Analysis

**FIGURE 6:**
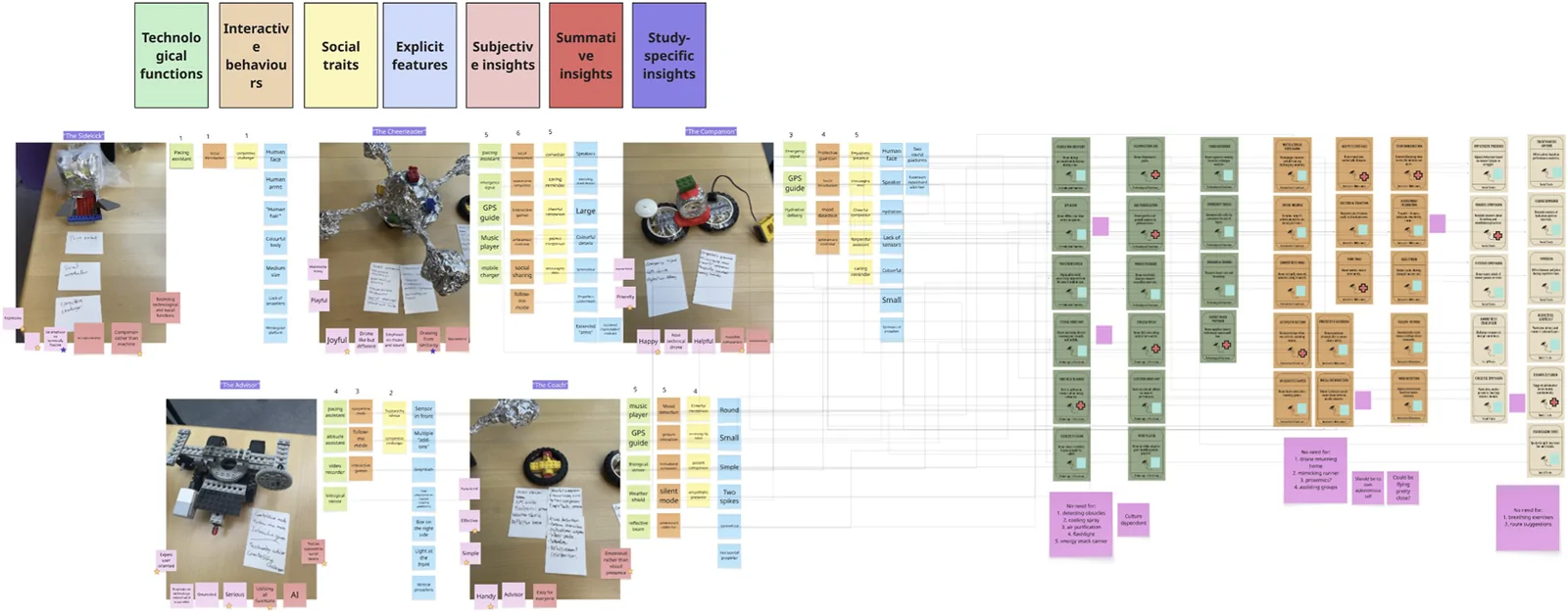
Annotated Visual Analysis of the drone prototypes and relation between the ideation cards and the drones.

The collected data were analyzed through two complementary approaches: a visual analysis and a deductive reflexive thematic analysis. The Annotated Visual Analysis (AVA) (Sturdee, 2025) was conducted by two researchers. AVA is a structured approach to identify relevant visual features of static images, such as sketches and photographs, complementary to what might be captured through verbal data collection methods. Following this approach, we identified *explicit features*, *summative insights*, *subjective insights*, and *study-specific insights* of the prototyped drones. Explicit features include discrete observations such as colors, size, and explicit anthropomorphic features, such as eyes and a mouth. Summative insights included an iteration where the researchers interpreted the implied meaning of the explicit features, for example, inferring that a smiling mouth means a helpful and approachable drone. Subjective insights follow a similar approach to inductive thematic analysis and, for this step, the researchers discussed the explicit features and summative features to identify insights across the five prototypes. This included insights such as some prototypes emphasizing technical features and other prototypes emphasizing social features. For the study-specific insights, the annotations were corroborated with the ideation cards, where we reviewed ways that the participants’ selected cards were reflected by the design. Figure 6 shows an overview of the AVA and the usage of the ideation cards for each drone in Miro.

The audio recordings were transcribed, using a university-supplied automated transcription service, followed by a manual correction of any errors. The transcripts were analyzed following the guidelines of Braun and Clarke (Braun and Clarke, 2024) on reflexive thematic analysis, as well as Gibbs’ standards of thematic coding and categorization (Gibbs, 2018). Our first iteration focused on descriptive coding and subsequent iterations focused on consolidating these into analytical themes. Three researchers listened to the full audio recording in a joint session while reading the transcripts. The transcripts were coded individually, starting from a set of predetermined codes [inspired by the Design Space for Social Drones framework by (Baytaş et al., 2019)]. These codes included *movement* (flight), *control methods*, *form*, *aesthetics*, *interaction modalities* (input/output), *social behaviors* (verbal/non-verbal), *appeal* (i.e., acceptance), *ergonomics*, *intentions*, *intuitive interaction*, *social role*, and *direct manipulation* (i.e., touch). In addition to these predetermined codes, we allowed for new codes where the findings in the data did not fit into the predetermined ones. Hence, the analysis team expanded the code list both individually and as a group through reflexive discussions during the coding session. The new codes were: *technical* (e.g., data storage), *purpose*, and *application* related to drone design concerns; and *ethics* and *non-user concerns* related to human-centered concerns. Using Miro, two of the co-authors formed themes from the codes during multiple discussion sessions, which were then analyzed through the lens of either the problem space or the solution space, with inspiration from the Social Robot Co-Design Canvases (Axelsson et al., 2022). In the following sections, the participants will be referred to as P1-P5.

### 4.2 Findings

Throughout the workshop, participants reflected on the use and design of drones to support recreational running. In this section, we present the low-fidelity prototypes produced by participants, including participants’ reflections tied to each prototype, followed by the analytical themes that demonstrate the necessity of extending and re-framing approaches to designing drones.

#### 4.2.1 Prototyped designs

**FIGURE 7:**
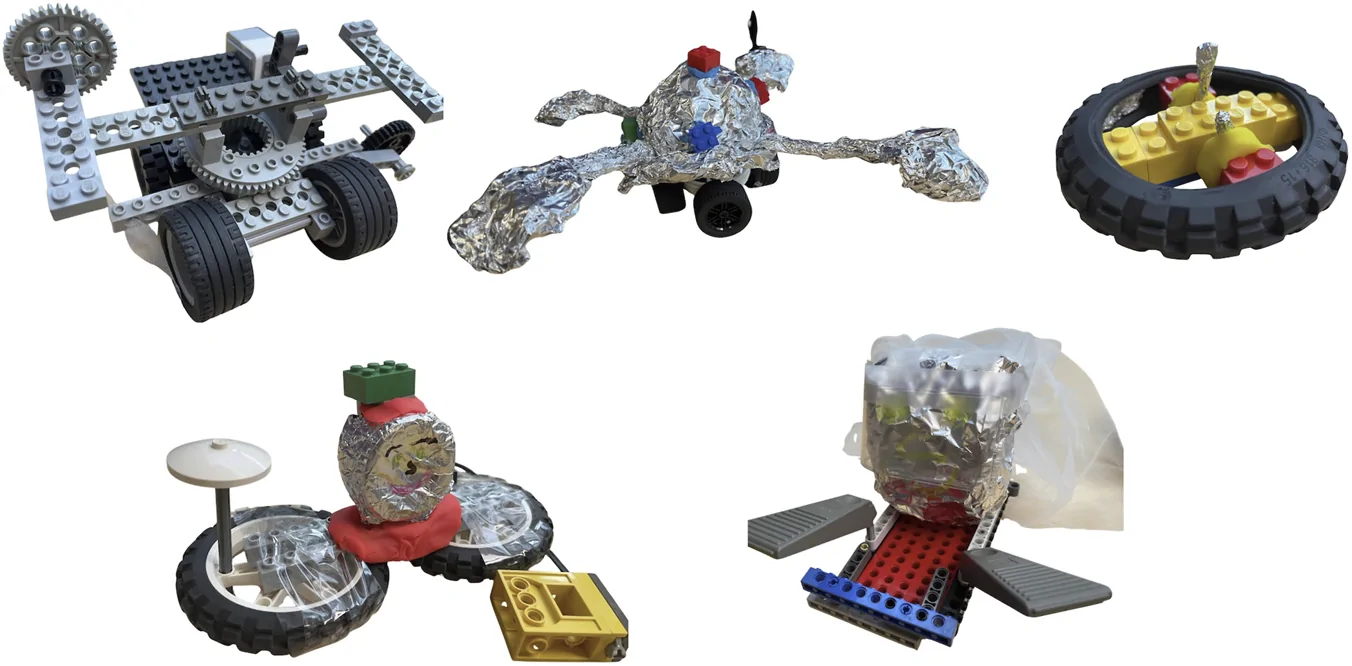
Low-fidelity prototypes of social drones for runners. From left to right, top to bottom: the drones were named ‘The Advisor’, ‘The Cheerleader’, ‘The Coach’, ‘The Companion’, and ‘The Sidekick’.

The drones created by the participants were *‘The Advisor’*, *‘The Cheerleader’*, *‘The Coach’*, *‘The Companion’*, and *‘The Sidekick’* (seen in Figure 7). Following the AVA, the prototypes exemplify a variety of distinct design decisions, supporting different goals derived from the personas and the environments in which they run. We emphasize here that these prototypes serve the purpose of eliciting what matters to recreational runners rather than representing product concepts.

The Advisor is the most technically capable drone, facilitating the need of accurate user data for advanced runners: *‘[the drone] is a full package of technology to record and measure when he [the persona] is running. There are a sensor and a heart rate monitor linked to his watch and shoes’* (P4). This drone focuses on improving numbers for its user, such as speed, distance and running style. The appearance reflects the focus on utility, designed in grayscale, and the role of this drone to be a tool and advisor. From the ideation cards, the design was expanded with interactive games and competitive mode, reflecting the personas focus on running results: *‘So [the drone] is recording him [the persona] and making noises, and maybe vibrations when he is not following the plan’* (P4). Also, as an expert on technology in their daily life, the drone is programmable with AI. In the ethical rankings, the advisor was considered one of the most ethically challenging drones since it collects both a lot of data of the user and potentially about bystanders without their knowledge.

The Cheerleader is multimedia heavy for recreational enjoyment, offering music and encouragements for runners who want to exercise to lower stress: *‘He [the persona] does social media a lot, so he needs good technology—but [he is] not the most competitive or the best like some others’* (P1). The design draws from conventional drone appearance but instead of propellers, the quadcopter has speakers to provide good sound quality: *‘[the drone] has speakers, because he [the persona] also feels alone but does not want to be social’* (P1). In the ideation cards it was reflected that this persona needed extra motivation to keep up with running by using the cards ‘cheerful’ and ‘patient companion’, ‘comedian’ and ‘encouraging voice’. In the ethical ranking, the Cheerleader was one of the least compromising since it did not collect any user data and did not try to mimic a relationship with the user, simply being an easy tool for encouragement.

The Coach is the smallest drone, being the size of a hand, and is designed to be a plug-and-play model that is simple and effective for anyone to use: *‘[the persona] is a very simple man, a practical man […] he is only interested in losing weight’* (P3). The user would receive support through reminders and planned runs to get the motivation to run more often to improve physical health. The ideation cards chosen were also practical: *‘the ‘GPS’ and the ‘weather shield’, especially, because when he [the persona] lacks motivation he does not go out. He definitely does not go out’* (P3).

The Companion is the drone with most social features. Its lack of technical features is compensated by emotional assistance during runs and friendly appearance: ‘[the drone] has a face and a speaker so it can talk, and it is in the air but just next to her [the persona]’ (P2). The drone is designed for users who enjoy running with company and might have a hard time keeping up with a running group’s schedule due to unsocial working hours: *‘the frustration was that people in the running clubs were too young, or that her [the personas’] working hours did not work with when the running club met. So I made her a drone to be her running buddy’* (P2). However, the ideation cards showed some technical functions, for example, the ‘hydration delivery’, which is visible in the prototype, but also: [the drone] have ‘GPS’ guide and ‘emergency signal’, because if she gets lost or if she falls, she is 60, so that would be very nice to have’ (P2). After a long discussion of the ethical considerations, the Companion was ranked the most ethically challenging drone since it is more personal, and personal information was ranked more intrusive than biometric data, such as running style.

The fifth and final drone is the Sidekick, designed as an anthropomorphic figure: *‘[the drone] is a bit of a character, it is like a floating head […] some human vibes’* (P5). This drone is physically expressive and balances utilitarian functions with social behaviors. The prototype itself is the least technically feasible (e.g., lacking visible propellers). Rather, the design emphasizes a joyful run for runners who mostly want something by their side. The ideation cards used for this prototype was ‘pacing assistant’, ‘competitive challenger’ and ‘social introduction’ which meant that the drone would help the user to find other runners mid runs. Like the Companion, the Sidekick was also considered a high risk during the reflection section since it mimics a human.

#### 4.2.2 Designing ‘social’ drones for running

Following the thematic analysis, we found that a social drone is not just social in a dyadic interaction with its user but rather situated in several social configurations. Beyond concerns for the *user* and the *drone*, the five drone designs and participants’ discussions also brought attention to concerns for *social relations*, *non-users*, and *communities*.

In terms of *social relations*, social drones can facilitate assistance between the user and people with whom there is already an established social relation, for example, a friend or family member. One participant said: ‘It can be of some kind of help if he [the persona] falls in the snow. It can signal something, maybe’ (P2). Another participant added: *‘[…] maybe for his [the persona’s] wife or something, if he is away for too long. She can see where he is’* (P3). Where social characteristics of the drone prototypes relating to the user were described in relation to the look of the drone, for example, the Sidekick having anthropomorphic features, facilitation of social relations emphasizes features such as technical reliability, far reach, and visibility. We summarize these perspectives in terms of *responsibility* and *assistance type*.

In terms of *non-users*, participants brought up external manipulation, consent, and malfunction. As one example of the bystander concerns, the workshop participants discussed the importance of data protection for bystanders where the drone could video record the user and, intentionally or not, also record other people. In the reflections phase, this was highlighted further, especially for the Advisor prototype, where the potential for data breaches of the drone would not only reveal data of the user but also about bystanders: *‘Maybe it could interfere or disturb others, like, let us say he [the persona] has a zone of something and the drone should stay within that, that maybe crosses other people’s paths in some way’* (P5). We summarize the participants’ perspectives in terms of *external manipulation* and *consent*.

For *communities*, the participants discussed how using a drone for running might affect how one is view (and potentially judged) by one’s community: *‘And maybe how people perceive him [the persona]. He is a priest, but he has this drone, so it is not like a stigma then’* (P3). Relating to proxemics, there is a relative position, not just relative to the user, but also depending on crowd behavior and physical environment. As, for example, with the Companion that is designed to fly very close to the user while, for example, the Advisor is adjusting the position to the task at hand. These drone behaviors can have different connotations by the people who observe them. We summarize these perspectives in terms of *non-user perspectives* and *crowd behavior*.

Finally, participants made comments relating to the ways the drones would navigate physical environments. They highlighted that the drones should not intrude on the surroundings, by, for example, hitting trees or disturbing a city environment. These concerns are important for the use case of running, as well as other drone applications in public spaces, where the drone should both adapt its role as running support while respecting the common space.

## 5 Discussion

Our results serve to validate and further detail aspects of social drone design captured in existing design frameworks (Baytaş et al., 2019). Considering Fang et al.’s Fang et al. (2026) categorization of theoretical frameworks, the Design Space for Social Drones framework (Baytaş et al., 2019) is both *descriptive* and *generative*. This means that the main value of the framework is to, first, provide a useful vocabulary through a categorization of phenomena, and second, to open a conceptual space that can inspire original ideas. The descriptive power of a framework can be justified based on how accurately and comprehensively it captures a phenomenon. Generative power cannot be validated but rather demonstrated through applying it to a concrete case. Thus, we consolidate our findings with the existing framework and discuss the utility of our social robot co-design approach in the context of running.

**FIGURE 8:**
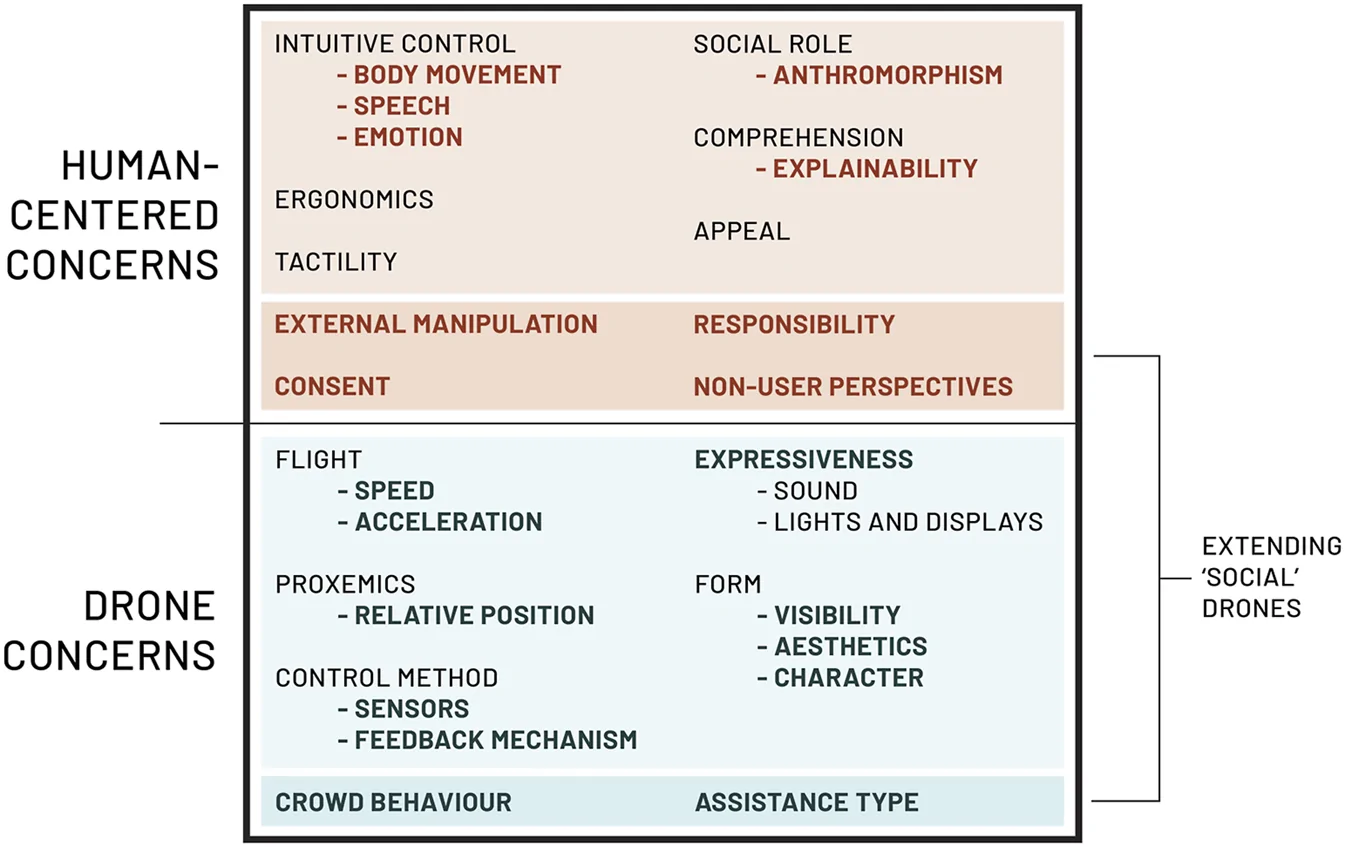
We extend the existing Design Space for Social Drones framework (Baytaş et al., 2019). Bold = Extension derived from the thematic analysis. Bottom of the two sections are non-user concerns.

While prior research (Axelsson et al., 2022) emphasizes that any design process needs to consider both the problem and the solution space, our findings further illustrate that design constraints relating to non-users, communities, and other social relations blur the lines between problem and solution. These design concerns at the same time constrain the problems and the solutions of social drones. On that basis, we suggest to add further details to the existing dimensions of the prior Design Space for Social Drones framework (Baytaş et al., 2019) as well as six additional design concerns, presented in Figure 8. The figure summarizes the added concerns of the design space and provides further parameters of dimensions from the original framework, e.g., that both *speed* and *acceleration* of a drone are parameters of designing the *flight* dimension. These parameters are derived from aspects highlighted by participants concerning their prototyped designs and other concerns relating to using social drones in a running context.

We propose that the Human-Centered Concerns should also cover non-user design concerns based on our thematic analysis, including *external manipulation*, *consent*, *responsibility*, and *non-user perspectives*. Furthermore, our analysis of the participants’ prototyped designs enable us to further detail the design concerns as follows:

- Intuitive control: The Companion and Cheerleader prototypes emphasized *speech* as a way of providing company and verbal encouragement. The prototypes also demonstrated a design concern relating to *emotion*, where some designs emphasized utility and others emphasized social behavior. Finally, participants emphasized the ways in which the drone moves according to the user, e.g., that a sidekick robot should mimic the ways that human running buddies would stay by the user’s side. We summarize this as *body movement.*
- Social role: A clear theme identified through the prototyped designs was the distinction between drones that mimic humans and drones that are used entirely for utilitarian purposes. As such, a design concern relating to the social role of the drone is the level of *anthropomorphism*, both in the physical design and the behavior of the drone.
- Comprehension: Particularly for the utilitarian prototyped designs, participants emphasized *explainability* as an important design concern. Thus, comprehension can encapsulate both understanding what a drone does as well as why.

In terms of Drone Concerns, our findings indicate that *crowd behavior* and *assistance type* are two crucial aspects of social drone design. Similar to the Human-Centered Concerns, we provide further details to the existing dimensions of the framework. Our findings suggest that the prior concerns of ‘sound’ and ‘lights and displays’ can be consolidated in a design concern relating to the *expressiveness* of a drone.

- Flight: Variable *speed* was a concern brought forth through the prototyped designs as a required feature for drones that need to fly alongside a human runner. As a related concern, acceleration can be useful in situations where a drone should incentivize the runner to increase their pace.
- Proxemics: The *relative position* between a drone and the user as well as other people in the environment is a concrete design concern relating to proxemics. Proximity may encapsulate other aspects of physical closeness, such as number of drones or humans in a space, and we add this design concern in service of extending the vocabulary.
- Control Method: Participants equipped the prototyped designs with multiple features to control drones. This included various imagined *sensors* as well as *feedback mechanisms.* We find this distinction crucial as control as a drone concern both encapsulates ways that the drone ‘sees’ the world and how users can confirm resulting actions.
- Form: We found that form can be distinguished in terms of *visibility*, *aesthetics*, and *character.* These design concerns speak to different goals of physical forms. Some participants emphasized the importance of visibility, particularly for non-users who may not be aware of the drone’s presence. For the prototyped designs, aesthetics and character played a role in how the drone supported the intended goal, e.g., to be a companion, participants imagined a drone as an entity with physical social characteristics.

We propose that our application and validation of the framework ultimately serve to extend the vocabulary of designing social drones. This supports the existing framework and offers actionable design concerns for future research.

### 5.1 Recommendations for social drone design

Prior research mapping design opportunities and challenges (Obaid et al., 2020a; Balasubramaniam et al., 2023b) reports on the constraints of the dyadic relationship between one user and one drone, with few exceptions such as (Baldursson et al., 2021; Johal et al., 2022), and addresses design processes as a linear process. Below, we list three design recommendations that encapsulate broader concerns in validation of the existing design framework.

Recommendation #1: Expose and filter contextual design concerns through co-design prototypes. Through a co-design workshop, we identified the unique roles drones could play—ranging from technical advisors to emotional companions—and the complex ethical, social, and contextual concerns that must be considered. Our proposed updated Social Drone Design framework integrates new stakeholders and design concerns, offering a more holistic lens for future drone design that can support runners. Overall, co-design proved valuable for navigating the under-explored space of social drone interactions, enabling imaginative yet grounded exploration of emerging technologies in running activities.

Recommendation #2: Balance anthropomorphic features with explainability. The updated framework highlights the importance of designing social drones that are both socially relatable and understandable. While anthropomorphic features can support engagement and trust in the running context for some users and non-users, ensuring that system behaviors remain transparent and explainable is critical to avoid misinterpretation. For others, where technical features come before social traits, explainability and responsibility become crucial for the drone design.

Recommendation #3: Emphasize perspectives of non-user stakeholders in the design. While the updated framework is grounded in the context of running; its principles extend to broader scenarios where drones operate in public spaces. Considering non-user stakeholders—such as bystanders and community members—ensures designs that respect diverse perspectives and social dynamics. Although tailored for running, these insights highlight the need to explore similar considerations in other activities, like cycling or hiking, thereby expanding the design space for social drones and guiding future work in varied physical and social contexts.

Furthermore, our findings highlight that designing social drones for recreational running cannot happen in a vacuum; it requires looking beyond the immediate runner-drone dyad. The discussion must extend to a wider ecosystem of peripheral stakeholders, including regulators, drone operators, and the general public. For instance, while a runner might prefer an autonomous, closely hovering companion, a member of the public sharing the path may view the exact same drone as an intrusive surveillance device or a safety hazard. Balancing these competing needs means that future HDI frameworks must account for non-user experiences, helping developers design systems that are socially acceptable not just to the primary user, but to the entire shared community.

### 5.2 Reflections on the co-design method

The familiarization phase with personas and scenes that were co-designed by the participant group, clearly established five different personas with diverse needs and challenges. Aided by the LEGO figures, they all followed through all subsequent phases and were articulated in the prototyped designs. Even though this phase was performed quite rapidly (15 min), they had a great impact on the whole workshop process. One thing that could be noted, is that even though the use of scenes was a short process that merely put the personas in a visual context, the participants ranked the scenes as the part that yielded most reflection and new ideas during the workshop and in the post workshop evaluation. To put the personas and drone usage into a space is one of the important parts of co-design, to not only develop a probable technology, but to imagine it in a future context.

In the brainstorming phase, the participants used the shared design space, and this introduced the idea of running with drones for the personas. By addressing the two first key insights from the preliminary study (difficulty to imagine drones for running and social drones overall) by prompting participants to discuss, for example, the role of the drone and its effect on society at large, making it possible for participants to think beyond these constraints. However, one recommendation for future use of a similar phase, is to allocate more time (we only had 20 min for this), since the participants wished to have longer detailed discussions for each of the personas.

The prototyping phase with the ideation cards elicited five different drone designs in regards of embodiment, technologies and sensors, as well as purposes and roles. All participants chose to use cards from all categories. However, there were some cards that were never used. For example, some cards could be interpreted as culture dependent, such as that the drone would aid with air purification or a cooling spray, which might be more relevant in warmer parts of the world. The cards that were used the most (by at least three participants) were those concerning technical functions for pacing and navigation, interactive behaviors for connecting runners mid run and during visual celebrations when milestones were reached, and cheering as a social trait.

The reflection phase was added to the workshop to address the third key insight from the preliminary study. The phase showed that ethics continues to be a big challenge with drone design and that it is a topic that elicits a lot of different viewpoints Altawy and Youssef (2016). For example, when it came to trusting the drone system, we could identify differences when it came to malfunctioning or misinterpreting data in how much users should generally trust a drone system that gives recommendations. The participants primarily considered it to be the user’s responsibility if something went wrong (e.g., if the user would get an injury after trusting recommendations). Another point was collection of body and emotional data, and for The Companion and The Sidekick, emotional data breaches could be more damaging to the user. One recommendation for future use of this phase is that the ranking of the designs in regard to handling ethical challenges might be too difficult since there often is no right or wrong answer, rather that all designs need to consider all possibilities, weigh them against each other, and in the end make their own choices.

### 5.3 Limitations

We acknowledge that a primary limitation of this study is its small sample size, which is a characteristic trade-off in qualitative design research. Rather than seeking statistical generalizability, our recruitment strategy was aligned with established qualitative standards that emphasize the richness, depth, and nuances of participants lived experiences, intentions, and conceptual strategies (Caine, 2016; Gibbs, 2018). Small, purposive samples allow for the deep, iterative exploration necessary to uncover latent user needs in emerging technology spaces, which is a recognized and well-supported approach (e.g., Vetter et al., 2024). That being said, our sample introduces a specific geographic and cultural boundary, as participants were recruited exclusively from two Western Nordic countries (Sweden and Denmark). The Nordic context is characterized by high levels of public trust in technology, specific outdoor recreational norms (such as the right to roam or so-called *allemansrätten* in Swedish), and distinct perceptions of public space privacy. Consequently, runners from other cultural, socio-economic, or geographic backgrounds may harbor entirely different preferences, anxieties, or expectations regarding drone design, safety, and social acceptability during recreational running. Future work should investigate how these design concepts translate to more diverse global contexts where public surveillance and recreational infrastructure differ.

Another limitation is the generalizability of the findings for other sports. As described in the Introduction, we selected recreational running as our central case of study since it is a ubiquitous, globally accessible activity requiring minimal resources, and because running presents a uniquely mature subfield within sports-drone literature. For these reasons, we found it suitable to practically evaluate the Design Space for Social Drones framework. However, our proposed modifications of the framework might not translate directly to drone applications within other sports.

A third limitation of our approach is that certain new codes (e.g., technical and ethics) may represent a byproduct of the workshop’s structured design rather than entirely spontaneous participant insights. Because the workshop explicitly guided participants through four structured phases using targeted design probes, there is an inherent risk of confirmation bias—where the data reflects the structural prompts we introduced. However, we argue that this structured scaffolding is a methodological necessity when engaging non-designers in co-design. Without intentional guidance, abstract or highly technical concepts like social drones can be difficult for participants to conceptualize. Probes and dedicated phases were essential to lower the barrier to entry and unlock meaningful discussion. Furthermore, the validity of this approach is supported by the fact that several new codes emerged entirely outside of this structural influence—such as non-user concerns—suggesting that the workshop left sufficient room for organic, unprompted insights.

For future research, we encourage others to draw on our proposed recommendations for designing and developing social drone applications through mixed methods approaches, such as experimental investigations with particular designs and in-situ investigations—both of which are pertinent next steps.

## 6 Conclusion

This paper investigated how social drones can be designed to support runners, contributing both a methodological approach and an updated design framework for future HDI systems for running. Three concrete design recommendations were developed based on eight interviews and a co-design workshop with five runners, where we sought engagement with diverse runner profiles (e.g., different ages, abilities, cultural backgrounds) to investigate concerns for social drone design. We encourage others to draw on our proposed recommendations for designing and developing social drone applications. *In-situ* investigations is a particularly pertinent next step in this endeavor.

## Data Availability

The datasets presented in this article are not readily available because of anonymisation of the participants. Requests to access the datasets should be directed to ST, sofia.thunberg@chalmers.se.

## Footnotes

1
https://www.bostonmagazine.com/health/2016/05/02/puma-beatbot
