# Supplementary material for: Investigating social drones for recreational running through co-design
Source: Front Robot AI. 2026 Sep 7;13:1897014. doi: 10.3389/frobt.2026.1897014 (PMC13590392; doi:10.3389/frobt.2026.1897014)
Supplement: Supplementary file 1 [file DataSheet1.pdf]

HYDRATION DELIVERY

Drone brings personalized hydration during runs.

Technological Functions

MOTIVATIONAL COMPANION

Encourages runners verbally during challenging moments.

Interactive Behaviours

EMPATHETIC PRESENCE

Adjusts behaviour based on runner fatigue or struggle.

Social Traits

GPS GUIDE

Drone offers real-time route navigation.

Technological Functions

SOCIAL SHARING

Streams runner's achievements live on social platforms.

Interactive Behaviours

MINDFUL COMPANION

Reminds runners about breathing and mindfulness practices.

Social Traits

WEATHER SHIELD

Deployable shield protecting runners from the sun or sudden rain.

Technological Functions

COMPETITIVE MODE

Drone virtually connects runners racing remotely.

Interactive Behaviours

PATIENT COMPANION

Drone waits calmly if runner pauses or rests.

Social Traits

ILLUMINATION AID

Drone illuminates' paths.

Technological Functions

ADAPTIVE DISTANCE

Drone maintains comfortable distance.

Interactive Behaviours

TRUSTWORTHY ADVISOR

Offers advice based on performance analytics.

Social Traits

AIR PURIFICATION

Drone purifies air around runners in polluted areas.

Technological Functions

GESTURE INTERACTION

Runners control drones easily via hand gestures.

Interactive Behaviours

CARING REMINDER

Reminds runners of hydration and rest intervals.

Social Traits

MOBILE CHARGER

Drone wirelessly charges runners' wearables mid-run.

Technological Functions

MIMIC MODE

Drone mimics runner movements.

Interactive Behaviours

COMEDIAN

Offers humour and jokes during repetitive runs.

Social Traits

VIDEO RECORDER

Drone captures running form for technique analysis.

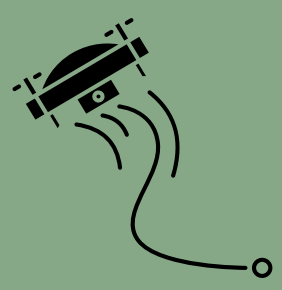

Technological Functions

TEAM COORDINATION

Drones help group runs to keep formations and pace.

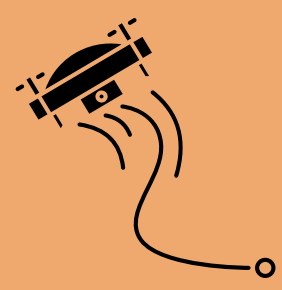

Interactive Behaviours

RESPECTFUL ASSISTANT

Maintains privacy and runners' personal space.

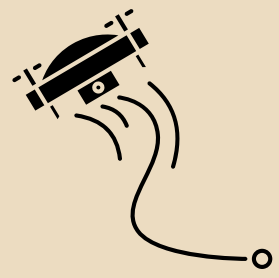

Social Traits

EMERGENCY SIGNAL

Automatically calls for assistance in case of injury.

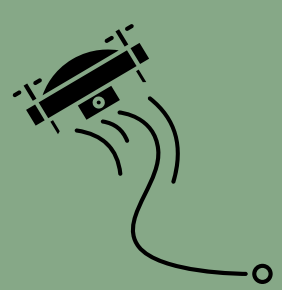

Technological Functions

ACHIEVEMENT CELEBRATOR

Visually celebrates milestones reached by runner.

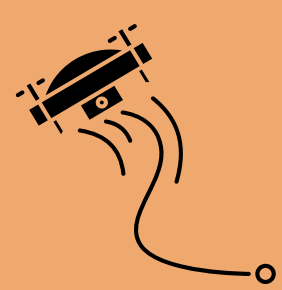

Interactive Behaviours

CURIOUS EXPLORER

Suggests alternative scenic routes spontaneously.

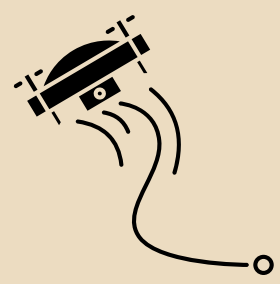

Social Traits

BIOLOGICAL SENSOR

Measures heart rate and breathing.

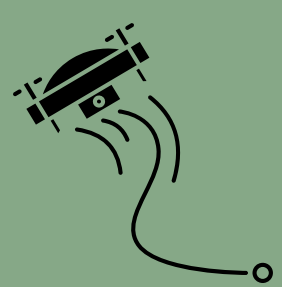

Technological Functions

SILENT MODE

Reduces noise during tranquil nature runs.

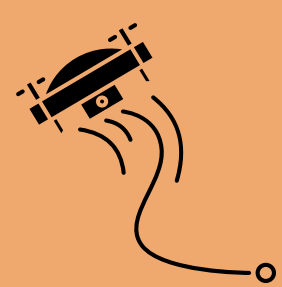

Interactive Behaviours

ENCOURAGING VOICE

Speaks in uplifting tones for motivation.

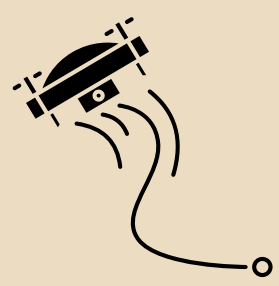

Social Traits

COOLING SPRAY

Drone delivers cooling mist in hot weather.

Technological Functions

FOLLOW-ME MODE

Automatically trails runners without direct commands.

Interactive Behaviours

COMPETITIVE CHALLENGER

Challenges runners to beat personal bests.

Social Traits

ALTITUDE ASSISTANT

Monitors altitude effects on runners' performance.

Technological Functions

MOOD DETECTION

Adjusts interactions based on runner emotions.

Interactive Behaviours

CHEERFUL COMPANION

Maintains positive presence, boosting runners' morale.

Social Traits

MUSIC PLAYER

Drone provides adaptive pace-matching music playlists.

Technological Functions

AUTOMATIC RETURN

Returns to base when not actively assisting runner.

Interactive Behaviours

PACING ASSISTANT

Drone maintains desired running pace visually and audibly.

Technological Functions

INTERACTIVE GAMES

Drone hosts interactive running games.

Interactive Behaviours

OBSTACLE SCANNER

Detects and warns runners of incoming obstacles.

Technological Functions

PROTECTIVE GUARDIAN

Drone positions strategically to ensure runner safety.

Interactive Behaviours

REFLECTIVE BEAM

Drone creates visibility beams at night for safety.

Technological Functions

SOCIAL INTRODUCTION

Drone facilitates social connections between nearby runners.

Interactive Behaviours

ENERGY SNACK PROVIDER

Drone supplies runners with energy snacks mid-run.

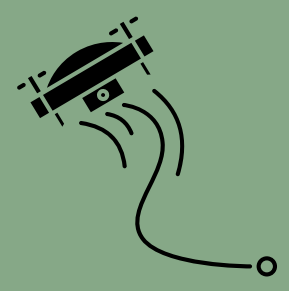

Technological Functions
